# Supplementary material for: Hybrid Models and Biological Model Reduction with PyDSTool
Source: PLoS Comput Biol. 2012 Aug 9;8(8):e1002628. doi: 10.1371/journal.pcbi.1002628 (PMC3415397; doi:10.1371/journal.pcbi.1002628)
Supplement: Text S4 — Complete source code for the PyDSTool package (version 0.88.120504). Includes API documentation and help files linking to web pages. This file is identical to the current public release on Sourceforge.net. (ZIP) [file pcbi.1002628.s004.zip › PyDSTool/html/PyDSTool.Toolbox.optimizers.step.newton_step-pysrc.html]

xml version="1.0" encoding="ascii"?


PyDSTool.Toolbox.optimizers.step.newton\_step


| Home | Trees | Indices | Help | PyDSTool |
| --- | --- | --- | --- | --- |

Package PyDSTool ::
Package Toolbox ::
Package optimizers ::
Package step ::
Module newton\_step

[hide private]

[frames] | no frames]

# Source Code for Module PyDSTool.Toolbox.optimizers.step.newton\_step

```
 1   
 2  # Matthieu Brucher 
 3  # Last Change : 2007-08-29 15:48 
 4   
 5  """ 
 6  Computes a Newton step for a specific function at a specific point 
 7  """ 
 8   
 9  import numpy 
10  import numpy.linalg 
11   


12 -class NewtonStep(object):


13    """ 
14    The Newton step 
15    """ 


16 -  def __call__(self, function, point, state):


17      """ 
18      Computes a Newton step based on a function and a point 
19      """ 
20      hessian = function.hessian(point) 
21      gradient = function.gradient(point) 
22      step = (-numpy.linalg.solve(hessian, gradient)).reshape(point.shape) 
23      state['hessian'] = hessian 
24      state['gradient'] = gradient 
25      state['direction'] = step 
26      return step

27
```

  


| Home | Trees | Indices | Help | PyDSTool |
| --- | --- | --- | --- | --- |

|  |  |
| --- | --- |
| Generated by Epydoc 3.0.1 on Fri May 4 15:24:25 2012 | http://epydoc.sourceforge.net |
